# Aloesin ameliorates hypoxic‐ischemic brain damage in neonatal mice by suppressing TLR4‐mediated neuroinflammation

**DOI:** 10.1002/iid3.1320

**Published:** 2024-06-18

**Authors:** Liping Chen, Siqing Xiong, Xiaofan Zhou, Qiang Fu

**Affiliations:** ^1^ Department of Rehabilitation Medicine Ji'an Central People's Hospital Ji'an Jiangxi Province China; ^2^ Department of Urinary Surgery Ji'an Central People's Hospital Ji'an Jiangxi Province China; ^3^ Department of Respiratory and Critical Care Medicine Ji'an Central People's Hospital Ji'an Jiangxi Province China; ^4^ Health Science Center Jinggangshan University Ji'an Jiangxi Province China

**Keywords:** Aloesin, hypoxic‐ischemic brain damage, neuroinflammation, TLR4

## Abstract

**Background:**

At present, neonatal hypoxic‐ischemic encephalopathy (HIE), especially moderate to severe HIE, is a challenging disease for neonatologists to treat, and new alternative/complementary treatments are urgently needed. The neuroinflammatory cascade triggered by hypoxia‐ischemia (HI) insult is one of the core pathological mechanisms of HIE. Early inhibition of neuroinflammation provides long‐term neuroprotection. Plant‐derived monomers have impressive anti‐inflammatory effects. Aloesin (ALO) has been shown to have significant anti‐inflammatory and antioxidant effects in diseases such as ulcerative colitis, but its role in HIE is unclear. To this end, we conducted a series of experiments to explore the potential mechanism of ALO in preventing and treating brain damage caused by HI insult.

**Materials and Methods:**

Hypoxic‐ischemic brain damage (HIBD) was induced in 7‐day‐old Institute of Cancer Research (ICR) mice, which were then treated with 20 mg/kg ALO. The neuroprotective effects of ALO on HIBD and the underlying mechanism were evaluated through neurobehavioral testing, infarct size measurement, apoptosis detection, protein and messenger RNA level determination, immunofluorescence, and molecular docking.

**Results:**

ALO alleviated the long‐term neurobehavioral deficits caused by HI insult; reduced the extent of cerebral infarction; inhibited cell apoptosis; decreased the levels of the inflammatory factors interleukin (IL)‐1β, IL‐6, and tumor necrosis factor‐α; activated microglia and astrocytes; and downregulated the protein expression of members in the TLR4 signaling pathway. In addition, molecular docking showed that ALO can bind stably to TLR4.

**Conclusion:**

ALO ameliorated HIBD in neonatal mice by inhibiting the neuroinflammatory response mediated by TLR4 signaling.

## Graphical abstract

1. Aloesin has neuroprotective effects in hypoxic‐ischemic brain damage. 2. The neuroprotective effect of Aloesin may be related to the inhibition of apoptosis caused by TLR4‐mediated neuroinflammatory response.

## 1 INTRODUCTION

Neonatal hypoxic‐ischemic encephalopathy (HIE) is one of the leading causes of brain damage and long‐term neurological dysfunction.^1^ Due to the improvements in neonatal resuscitation techniques, the hazards of neonatal HIE in developed countries have been reduced, but in developing countries, the incidence of neonatal HIE remains high, even reaching 26%.^1^ Moderate and severe HIE easily leads to cerebral palsy, epilepsy, cognitive impairment, emotional disorders, learning disabilities, and directly leads to the death of neonates, seriously affecting the quality of life of patients and their families and imposing additional burdens on society.^2^, ^3^ Therefore, to minimize or even prevent the above adverse consequences, it is necessary to actively intervene in HIE. Unfortunately, there is still no effective treatment for HIE. At present, mild hypothermia therapy is considered the only routine treatment for this disease.^4^, ^5^ However, studies have shown that mild hypothermia therapy not only fails to reduce HIE patient mortality or disability at 18 months but also significantly increases the mortality of HIE patients in low‐ and middle‐income countries.^6^ Therefore, the development of new alternative or complementary treatments needs to be accelerated to maximize the relief of neonatal HIE.

Hypoxia‐ischemia (HI) causes excessive neuroinflammatory responses that exacerbate brain damage.^7^ It also causes a reduction in or interruption of blood flow and oxygen supply, neuronal energy depletion, and death. Moreover, HI injury strongly activates brain‐resident immune cells, such as microglia and astrocytes, and induces the infiltration of circulating peripheral leukocytes and immune cells.^8^, ^9^ Activated immune cells release large amounts of proinflammatory factors, such as interleukin (IL)−1β, IL‐6, and tumor necrosis factor (TNF)‐α, which further lead to secondary neuronal damage.^8^ When the neuroinflammatory cascade is blocked in the early stage, brain damage is reduced. Many studies have shown that regulating neuroinflammation can effectively ameliorate brain damage caused by HI insult and ameliorate neurological deficits. Therefore, neuroinflammation is expected to become a potential target for the treatment of HIE.

**Figure 1:**
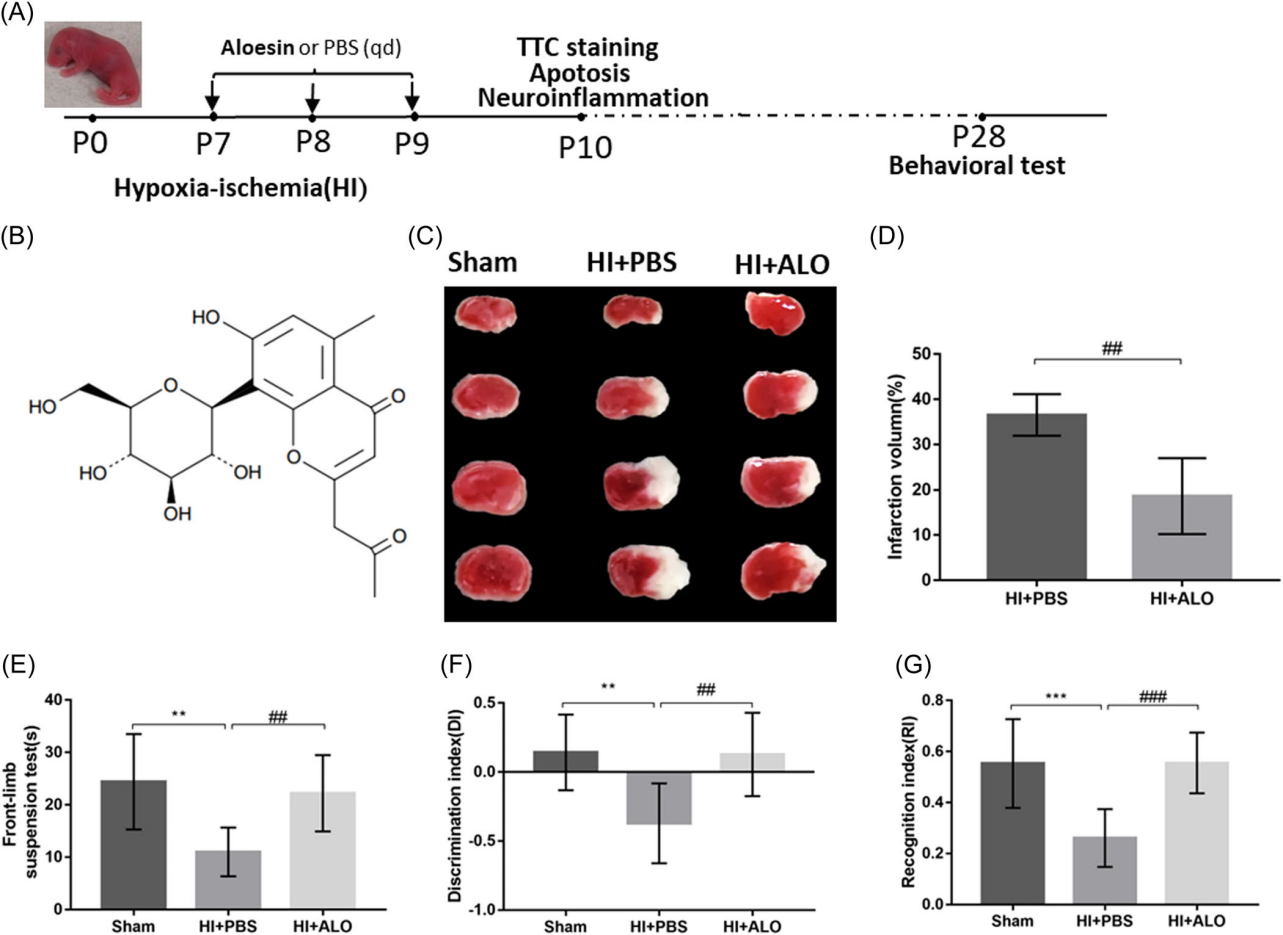
Effects of aloesin on cerebral infarct size and neurobehavior in HIBD mice. (A) Schematic diagram of the experiment. (B) The chemical structure of aloesin. (C) Representative images of TTC‐stained slices of brain. (D) Quantification of the infarct size as a percent of the contralesional hemisphere (*n* = 6 in each group). (E) Latency of mice to fall from the glass rod in the forelimb suspension test (*n* = 8 in each group). (F) The discrimination index in the novel object recognition test (*n* = 8 in each group). (G) The recognition index in the novel object recognition test (*n* = 8 in each group). Statistical significance was determined by Student's *t* test. The values are expressed as the mean ± standard deviation: *^,#^ *p* < .05, **^,##^ *p* < .01, ***^,###^ *p* < .001. ALO, Aloesin; HI, hypoxia‐ischemia; HIBD, hypoxic‐ischemic brain damage; PBS, phosphate‐buffered saline; TTC, 2,3,4‐triphenyltetrazolium chloride.

In recent years, traditional Chinese medicine monomers extracted from natural plants have been used to treat hypoxic‐ischemic brain damage (HIBD) model rodents and have shown positive effects due to their rich sources and multitarget effects, such as resveratrol,^10^ curcumin,^11^ and quercetin.^12^ The chemical name of Aloesin (ALO) is 2‐acetonyl‐8‐β‐d‐glucopyranosyl‐7‐hydroxy‐5‐methylchromone. Its structural formula is shown in Figure 1B. Its molecular formula is C_19_H_22_O_9,_ its molecular weight is 394.13, and its aloe vera content is approximately 3‰.^13^ ALO has been shown to have antioxidant and anti‐inflammatory properties and has been mostly used in skin and cosmetic medicine studies,^14^, ^15^ but its role in HIE has not been reported thus far. Therefore, in the present study, we administered ALO to neonatal HIBD model mice to explore whether this treatment could provide neuroprotection and the potential underlying mechanism.

## 2 MATERIALS AND METHODS

### 2.1 Experimental animals and HIBD model construction

Institute of Cancer Research (ICR) male and female mice were purchased from Hunan STA Laboratory Animal Co., Ltd. and paired with one male and two females for cocaging. Pregnant mice were separated into separate cages before delivery, and the time of delivery was recorded to determine the exact age of the newborn mice. When the newborn mice were 7 days old, they were used for HIBD model generation, regardless of sex. Briefly, after the neonatal mice were anesthetized with isoflurane, they were fixed on the operating table with medical tape. The alcohol‐sterilized front neck skin was cut from the center. Blunt‐tipped forceps were used to slowly separate the muscles layer by layer, and the right common cervical cartilage was found next to the trachea. The common carotid artery often accompanies the vagus nerve. After the vagus nerve was carefully separated, the common carotid artery was ligated with 6.0 surgical thread. Finally, the skin was sutured. After the pups woke up and resumed normal breathing, they were returned to the cage and allowed to recover for 1 h. The postoperative mice were then transferred to a hypoxic box. The temperature of the hypoxic box was set to 37°C, and mixed hypoxic gas (8% oxygen + 92% nitrogen) was continuously introduced at 1 L/min for a total of 1 h. The sham group was not subjected to arterial ligation or hypoxia. Mice in the HI+PBS group received injections of vehicle only. Mice in the HI+ALO group were intraperitoneally injected with 20 mg/kg aloesin (Zhongshan CN‐Biotechnology Co., Ltd.) at three time points: immediately, 24 h, and 48 h after the end of hypoxia. In addition, we established a sham group treated with ALO to observe the effect of ALO on normal mice, and the results are provided in the supplementary material. Animal studies were conducted in accordance with the National Institute of Health Guide for the Care and Use of Laboratory Animals and reported in compliance with the ARRIVE guidelines. The design of the experiment is shown in Figure 1A.

### 2.2 Measurement of infarct size

The cerebral infarction volume is one of the key indicators used to evaluate the degree of brain damage. We used 1% 2,3,4‐triphenyltetrazolium chloride (TTC) solution to stain coronal slices of fresh brain tissue. TTC staining was performed 72 h after HI, when the mice were 10 days old. The slice thickness was 1 mm, and the reaction time was 20 min. The ischemic infarction area was stained white, and the normal brain tissue was stained red. ImageJ software was used to measure the infarct area and calculate the ratio of the infarct area to the total brain area.

### 2.3 Neurobehavioral tests

#### 2.3.1 Novel object recognition (NOR) test

The NOR test was used to assess cognitive function. The NOR test is based on the degree to which mice are more interested in novel objects than familiar objects. An open field was prepared, and a camera located above the center of the field was used to record and analyze the behavioral trajectory of the mice. Mice were placed in the arena in advance to familiarize themselves with the testing area for 10 min and then allowed to explore two identical objects that were subsequently placed in the arena for 5 min. After 24 h, one of the objects was replaced with a new object that had a different material and shape than those of the original object. The animal was put into the field again to explore, and the time spent exploring the novel and old objects was recorded. The discrimination index (DI) and recognition index (RI), which are memory measures that assess cognitive function, were calculated. DI = (TN − TF)/(TN + TF); RI = TN/(TN + TF); TF = time spent interacting with the familiar object; TN = time spent interacting with the novel object.

#### 2.3.2 Forelimb suspension test

The forelimb suspension test was used to evaluate the muscle strength and motor coordination of the mice. The mouse was placed on a smooth glass rod 45 cm high, the timer started when the mouse grasped the glass rod with its forelimbs, and the timer ended when the mouse dropped. A shorter time on the glass rod indicated poorer muscle strength and motor coordination.

### 2.4 Terminal deoxynucleotidyl transferase dUTP nick end labeling (TUNEL) staining

TUNEL staining was used to detect nuclear DNA fragmentation during the process of cell apoptosis. A TUNEL assay kit (#KGA7071; KeyGEN) was purchased, and the specific steps were carried out according to the product manual. Briefly, mouse brain cryosections fixed with 4% paraformaldehyde were permeabilized with proteinase K solution, and then TdT enzyme reaction solution was added and incubated in the dark for 1 h. After completing the above steps, the brain slices were rinsed with phosphate‐buffered saline (PBS), and 50 μL of streptavidin‐fluorescein labeling solution was added to the sections, which were subsequently incubated at 37°C for 30 min. The final step involved staining the nuclei with DAPI (4′,6‐diamidino‐2‐phenylindole) and observing the sections under a confocal laser microscope.

### 2.5 Western blot analysis

A special extraction kit (KeyGEN Biotec) was used for total protein extraction, and the ipsilateral hippocampus (HI) was homogenized and lysed in lysis buffer containing radioimmunoprecipitation assay buffer, protease inhibitors, and phosphatase inhibitors with manual shaking three times during the process. After low‐temperature centrifugation, the supernatant was collected to determine the protein concentration via the bicinchoninic acid assay method. Sodium dodecyl‐sulfate polyacrylamide gel electrophoresis was used to separate the proteins, after which the proteins were transferred to a polyvinylidene fluoride (PVDF) membrane, which was subsequently blocked with 5% nonfat milk solution. The PVDF membranes were incubated overnight at 4°C with the corresponding primary antibodies. The next day, the membranes were incubated with horseradish peroxidase‐conjugated secondary antibodies, and an enhanced chemiluminescence (ECL) substrate was applied for protein detection. Gray value analysis was performed using ImageJ software.

### 2.6 Quantitative real‐time polymerase chain reaction

Total RNA was extracted from the ipsilateral HI using TRIzol reagent from Life Technologies, after which the optical density of the extracted RNA was measured. If the optical density was between 1.8 and 2.2, the sample was considered usable, 1 μg of available RNA was reverse‐transcribed into 20 μL of cDNA, and specific primers and SYBR PCR Master were used to amplify 2 μL of the synthesized cDNA. The kit used for reverse transcription and amplification was obtained from Vazyme, and β‐actin was used as an internal reference. The values of the target genes were normalized to the fold changes determined using the 2^−ΔΔ*C*^T method. The sequences of primers used were as follows: IL‐1β forward, 5′‐GAAATGCCACCTTTTGACAGTG‐3′; IL‐1β reverse, 5′‐ TGGATGCTCTCATCAGGACAG‐3′; IL‐6 forward, 5′‐GCTGGTGACAACCACGGCCT‐3′; IL‐6 reverse, 5′‐AGCCTCCGACTTGTGAAGTGGT‐3′; TNF‐α forward, 5′‐CAAGGGACAAGGCTGCCCCG‐3′; TNF‐α reverse, 5′‐GCAGGGGCTCTTGACGGCAG‐3′; β‐actin forward, 5′‐TTCTTGGGTATGGAATCCTGT‐3′; and β‐actin reverse, 5′‐AGCACTGTGTTGGCATAGAG‐3′.

### 2.7 Immunofluorescence staining

The ipsilateral HI served as the examination area. Briefly, after cardiac perfusion of normal saline and 4% paraformaldehyde, the brains of the mice were immersed in 4% paraformaldehyde overnight. The brains were then gradient dehydrated using 20% and 30% sucrose solutions, after which cryosections were cut. The cryosections were incubated with primary antibodies overnight at 4°C followed by incubation with Alexa Fluor‐conjugated secondary antibodies in the dark. DAPI was used to visualize the cell nuclei. A confocal laser microscope was used to acquire images of the sections.

### 2.8 Molecular docking method

Molecular docking was performed to assess the binding affinity between the compounds and their protein targets. The structures of the protein targets were acquired from the RCSB protein database (PDB) (http://www.rcsb.org/),^16^ and the crystal structure of ALO was downloaded from the PubChem database (https://pubchem.ncbi.nlm.nih.gov/) as an SDF file and converted into mol2 format using Chimera. The target protein was used as the grid center, while the center coordinate (center *x*/*y*/*z*) and box size (size *x*/*y*/*z*) were adjusted to cover the protein completely. Molecular docking simulations were carried out using AutoDock Vina (https://vina.scripps.edu/).^17^ After docking with Vina, the scores for the combinations of proteins and small molecules were calculated, and PyMOL and Discovery Studio software were used to perform force analysis and visualization from three‐dimensional and two‐dimensional perspectives, respectively.

### 2.9 Statistical analysis

The available data obtained from the experiments are expressed as the mean ± standard deviation, and each experiment was repeated at least three times. SPSS20.0 software was used for statistical analysis, and GraphPad Prism 7.0 software was used for graphing. Student's *t* test (double‐tailed) was used for data comparisons between two groups, while one‐way analysis of variance and the least significant difference post hoc test were used for data comparisons between three groups or more. A *p* value less than .05 was considered to indicate a significant difference.

## 3 RESULTS

### 3.1 Aloesin restricts HI insult‐induced infarct size and neurobehavioral deficits

There were no significant differences in body weight or neurobehavioral performance between the sham group and the sham group administered ALO (Figure S1). Compared with those in the sham group, the cerebral infarct areas in the HI insult group were significantly greater (Figure 1C,D). Moreover, HI insult also caused obvious neurological deficits, as indicated by a faster fall from the glass rod (Figure 1E), which indicated decreased limb coordination on both sides and weakened strength of the affected forelimb. The DI and RI were also lower in the experimental group than in the sham group (Figure 1F,G), indicating a decrease in the ability to distinguish old and new objects. After the administration of ALO, the cerebral infarct volume was reduced, and the neurobehavioral deficits were significantly reversed.

### 3.2 Aloesin inhibits HI insult‐induced neuronal apoptosis

**Figure 2:**
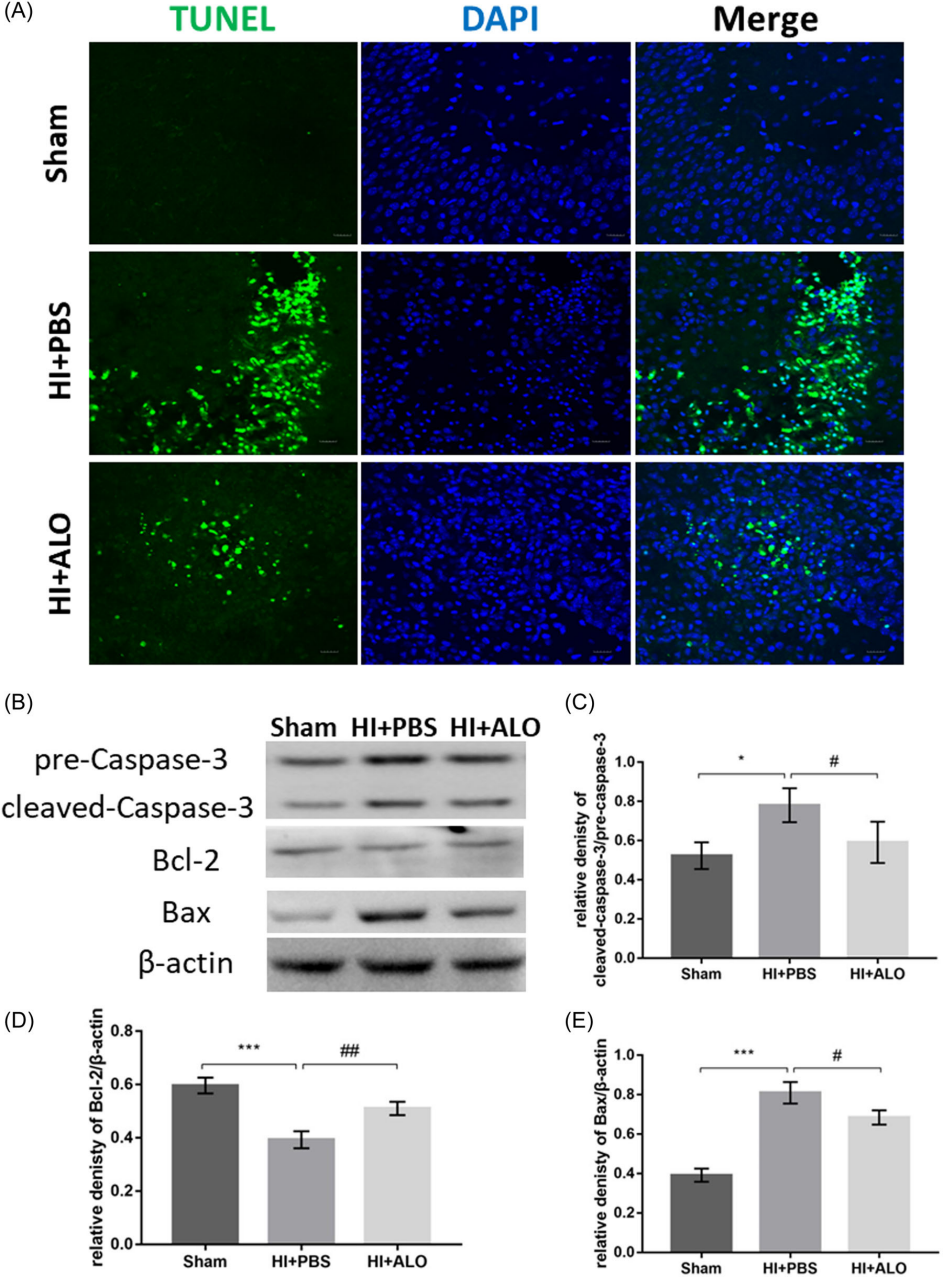
Effect of aloesin on neuronal apoptosis in HIBD mice. (A) Representative images of TUNEL‐staining. Scale bar, 100 μm. *n* = 3 in each group. (B) Representative western bands for apoptosis‐related proteins Bax, Bcl‐2, and cleaved caspase‐3. (C)–(E) Densitometry quantification of cleaved caspase‐3 (C), Bcl‐2 (D), and Bax (E). *n* = 4 in each group. Statistical significance was determined by one‐way analysis of variance followed by the least significant difference multiple comparisons test. The values are expressed as the mean ± standard deviation: *^,#^ *p* < .05, **^,##^ *p* < .01, ***^,###^ *p* < .001. ALO, Aloesin; DAPI, 4′,6‐diamidino‐2‐phenylindole; HI, hypoxia‐ischemia; HIBD, hypoxic‐ischemic brain damage; PBS, phosphate‐buffered saline; TUNEL, terminal deoxynucleotidyl transferase dUTP nick end labeling.

TUNEL staining revealed that HI insult caused a significant increase in TUNEL‐positive cells (Figure 2A). Moreover, the expression of apoptosis‐related proteins changed (Figure 2B), among which the expression levels of Bax and cleaved caspase‐3 increased (Figure 2C,E) and those of bcl‐2 decreased (Figure 2D), indicating that HI insult promoted apoptosis, while the application of ALO decreased the number of TUNEL‐positive cells and mitigated the abnormal expression of apoptotic proteins.

### 3.3 Aloesin downregulates HI insult‐induced inflammatory factor expression and reduces glial activation

**Figure 3:**
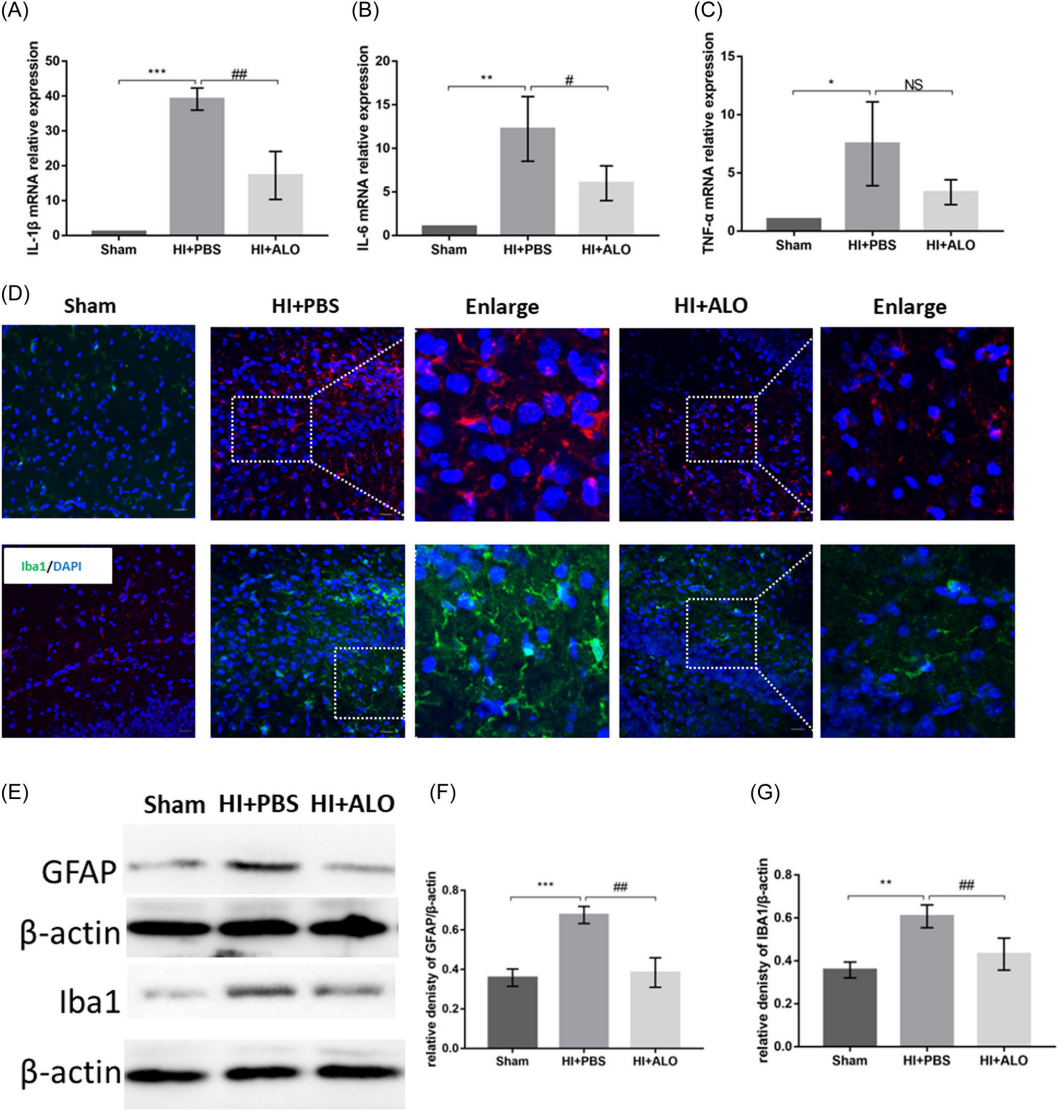
Effects of aloesin on inflammatory factors and glial activation in HIBD mice. (A)–(C) The messenger RNA expression of inflammatory factors IL‐1β (A), IL‐6 (B), and TNF‐α (C). *n* = 4 in each group. (D) Representative immunofluorescence images of GAFP‐positive astrocytes (red) and iba1‐positive microglia (green). *n* = 3 in each group. (E) Representative western bands for GFAP and iba1. (F) and (G) Densitometry quantification of GFAP (F) and iba1 (G). *n* = 4 in each group. Statistical significance was determined by one‐way analysis of variance followed by the least significant difference multiple comparisons test. The values are expressed as the mean ± standard deviation: NS, *p* > .05, *^,#^ *p* < .05, ^**,##^ *p* < .01, ^***,###^ *p* < .001. ALO, Aloesin; GFAP, glial fibrillary acidic protein; HI, hypoxia‐ischemia; HIBD, hypoxic‐ischemic brain damage; NS, not significant; PBS, phosphate‐buffered saline.

Inflammation‐related indicators (IL‐1β, IL‐6, and TNF‐α) were observed, and while HI insult led to an increase in the levels of these inflammatory mediators, ALO treatment limited this increase (Figure 3A–C). In addition, HI insult also resulted in the enhanced fluorescence intensity (Figure 3D) and upregulated protein expression (Figure 3E–G) of specific markers of astrocytes (glial fibrillary acidic protein [GFAP]) and microglia (iba1) in the brain. Similarly, ALO prevented these changes.

### 3.4 Aloesin restrains HI insult‐induced activation of TLR4 signaling

**Figure 4:**
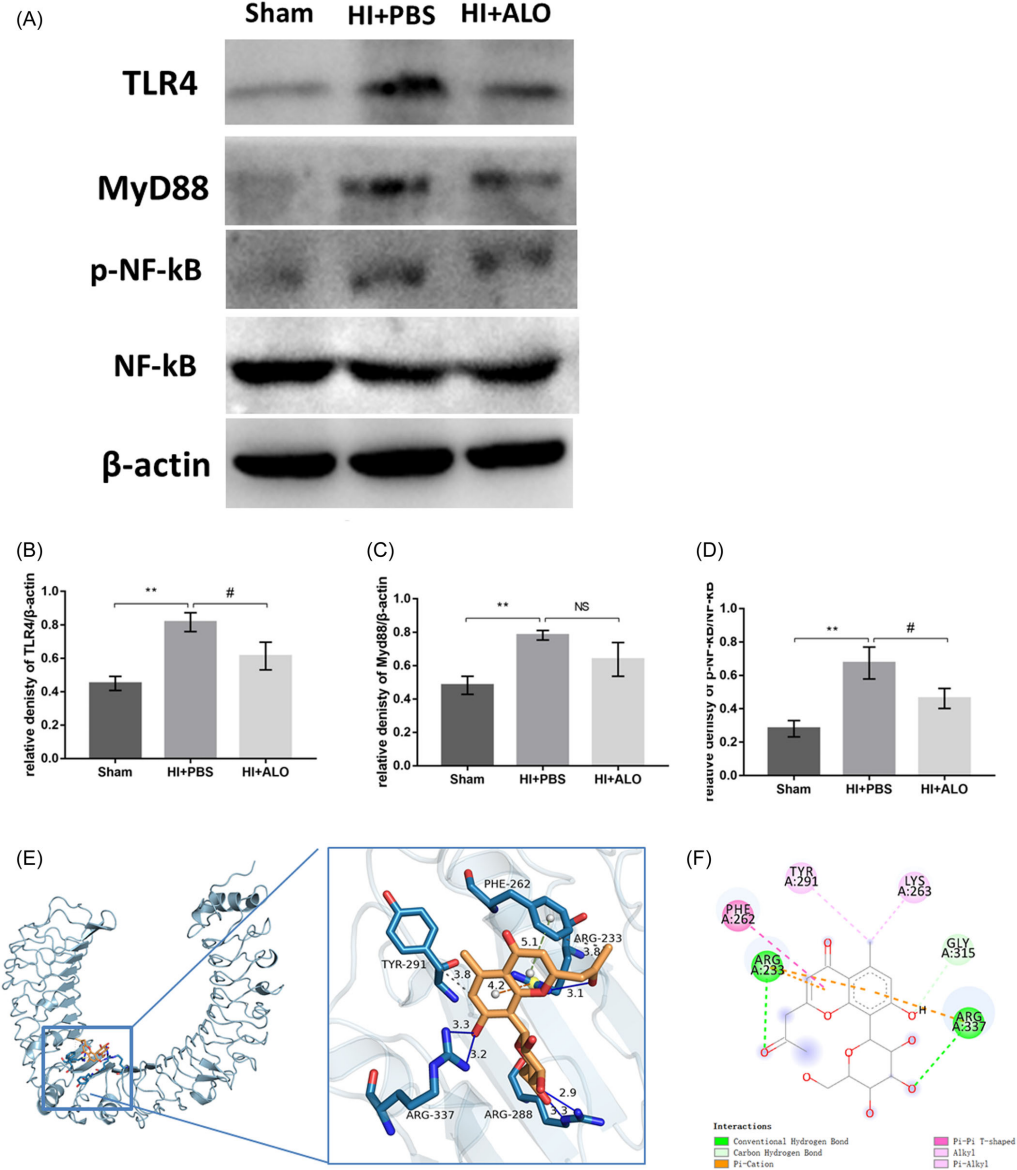
Effects of ALO on TLR4/MyD88/NF‐κB signaling pathway in HIBD mice. (A) Representative western bands for TLR4, MyD88, and NF‐κB. (B)–(D) Densitometry quantification of TLR4 (B), MyD88 (C), and NF‐κb (D) (*n* = 4 in each group). (E) Molecular docking site and three‐dimensional force analysis diagram of ALO and TLR4. (F) Two‐dimensional force analysis diagram of ALO and TLR4 (*n* = 4 in each group). Statistical significance was determined by one‐way analysis of variance followed by the least significant difference multiple comparisons test. The values are expressed as the mean ± standard deviation: NS, *p* > .05, *^,#^ *p* < .05, **^,##^ *p* < .01, ***^,###^ *p* < .001. ALO, Aloesin; HIBD, hypoxic‐ischemic brain damage; NS, not significant.

Since ALO can inhibit the neuroinflammatory response caused by HI insult, we conducted further experiments and found that HI insult upregulated the expression of key proteins in the Toll‐like receptor 4 (TLR4)/myeloid differentiation primary response gene 88 (Myd88)/nuclear factor kappa‐B (NF‐κB) signaling pathway, and ALO treatment reduced the abnormally elevated protein expression levels (Figure 4A–D). The binding activity of ALO to TLR4 was verified through molecular docking, and the binding energy between ALO and the TLR4 protein was found to be −6.5 kcal/mol. The three‐dimensional diagram shows that ALO interacts through hydrogen bonds, cation‐π interactions, and hydrophobic forces and that the Π–Π stacking force binds to the five amino acid residues of the TLR4 protein (Figure 4E). In addition, two‐dimensional force analysis yielded similar results (Figure 4F).

## 4 DISCUSSION

Under harmful stress conditions, such as insufficient oxygen or/and blood flow, a sterile neuroinflammatory response occurs in the central nervous system. This uncontrolled neuroinflammation can lead to secondary damage to neurons. Since the neonatal brain is in a stage of rapid development, this pathological phenomenon is particularly obvious in neonatal HIE and leads to generalized neurological dysfunction. To clarify whether ALO has a neuroprotective effect, we first evaluated cerebral infarction and neurobehavioral changes. We first found that ALO can reduce the volume of cerebral infarction caused by HI insult and that ALO also improves limb coordination, muscle strength, and cognitive function. Additionally, ALO has no adverse effects on normal mice. The above results demonstrated that ALO is safe for neonatal mice and has a definite neuroprotective effect on neonatal HIBD model mice.

ALO is an active ingredient isolated from *aloe vera* and is among the most common chromones. Lucini et al. reported that the 5‐methylchromone ALO is one of the most active metabolites among the pure secondary metabolites tested.^14^ Several studies have confirmed that ALO has suitable antioxidant and anti‐inflammatory effects. In ulcerative colitis models, ALO reduces LTB4 and TNF‐α levels and inhibits myeloperoxidase activity and TNF‐α and IL‐1β mRNA expression.^15^ In addition, ALO inhibited LPS‐induced COX‐2 gene expression in macrophages, so the inhibitory effect of the ALO diet on plasma PGE2 levels may be based on the inhibition of the COX‐2 pathway.^18^, ^19^ The present study revealed for the first time that ALO also has a significant neuroprotective effect, at least in the HIBD model. These neuroprotective effects may be achieved mainly by reducing nerve cell apoptosis triggered by HI insult. Further research revealed that ALO can inhibit the HI insult‐induced expression of IL‐1β, IL‐6, and TNF‐α to block excessive activation of the neuroinflammatory response and suppress the overactivation of microglia and astrocytes.

Neuroinflammation is one of the most important pathophysiological mechanisms of HIBD. Since we first discovered that ALO can inhibit the inflammatory response induced by HI, we continued to explore possible targets of ALO. We found that the upregulation in TLR4 expression induced by HI insult was reduced after the administration of ALO. In the brain, TLR4 is expressed mainly on microglia and astrocytes, and many endogenous and exogenous stimuli induce a proinflammatory response by activating TLR4 on glial cells^20^ and further guide various types of cell necrosis and apoptosis in the central nervous system.^21^ If TLR4 signaling is incorrectly activated or amplified uncontrollably, excessive production of cytokines may have a destructive effect on the nervous system.^22^ Therefore, TLR4 is widely involved in a variety of neurological diseases, such as cerebral infarction,^23^ cerebral hemorrhage,^24^ neurodegeneration,^25^ and mood disorders, including depression,^26^ and TLR4‐deficient mice exhibit improvements in neurological and/or behavioral outcomes in various cerebral infarction models.^27^ A series of studies have also reported that the TLR4/MyD88/NF‐κB signaling pathway plays an important pathophysiological role in HIE rodent models. The use of the TLR4‐specific antagonist TAK‐242 significantly reduces the brain damage and neurobehavioral defects caused by HI insult.^28^ Furthermore, some natural compounds, such as resveratrol, glycyrrhizin, and quercetin, also exert neuroprotective effects on HIBD model mice by regulating the upstream and downstream signaling pathways of TLR4.^29^, ^30^, ^31^ Therefore, TLR4 is an important pathological molecule in neonatal HIBD. In addition to experimental verification, we also used molecular docking methods to demonstrate the relationship between ALO and TLR4. The results of molecular docking intuitively demonstrated the occurrence of different kinds of forces between ALO and various amino acid residues at different sites on TLR4, such as ARG, TYR, and PHE; these forces ensure that ALO and TLR4 can bind stably.

Although we conducted a series of experiments to support the therapeutic effects of ALO, there are still some limitations. We only analyzed TLR4 expression in the total brain tissue protein while previous studies on HIBD have focused mainly on microglial TLR4.^30^ Since TLR4 is expressed on microglia and astrocytes, TLR4 expression on astrocytes in the context of HIBD needs to be further explored. In addition, in the future, TLR4‐specific inhibitory lentiviruses should be injected into the brain areas most susceptible to HI insult, such as the cortex and hippocampus, to further clarify the neuroprotective mechanism of ALO in the context of HIBD. Finally, other inflammatory signaling pathways are involved in the HIBD process. However, whether ALO works by inhibiting other inflammatory signaling pathways requires further in‐depth exploration.

## 5 CONCLUSION

In summary, ALO may reduce HI insult‐induced apoptosis and cerebral infarction by inhibiting glial cell activation and neuroinflammation mediated by the TLR4/MyD88/NF‐κB signaling pathway, thereby improving long‐term neurological function. These findings expand the disease treatment potential of ALO, provide new complementary insights into the anti‐inflammatory mechanism of ALO, and provide a new direction for the treatment of neonatal HIE.

## AUTHOR CONTRIBUTIONS

Liping Chen conceived and designed the study. Siqing Xiong and Qiang Fu conducted the experiments. Liping Chen drafted the initial manuscript. Xiaofan Zhou edited and revised the whole manuscript. All authors reviewed and contributed to the final version of the manuscript.

## CONFLICT OF INTEREST STATEMENT

The authors declare no conflict of interest.

## ETHICS STATEMENT

All experiments were approved by the Ethical Committee of Ji'an Central Hospital (Ethics No. (2023)030602).

## Supporting information


**Fig. S1. Effects of aloesin on normal mice**. A. Changes in mouse body weight at different time points (n = 8 in each group). B. The discrimination index in the novel object recognition test (n = 8 in each group). C. The recognition index in the novel object recognition test (n = 8 in each group). D. Latency of mice to fall from the glass rod in the forelimb suspension test (n = 8 in each group). Statistical significance was determined by Student's t test. The values are expressed as the mean ± standard deviation: ns, *p* > 0.05.
