# Supplementary figures and images for: Aloesin ameliorates hypoxic‐ischemic brain damage in neonatal mice by suppressing TLR4‐mediated neuroinflammation
Source: Immun Inflamm Dis. 2024 Jun 18;12(6):e1320. doi: 10.1002/iid3.1320 (PMC11184644; doi:10.1002/iid3.1320)

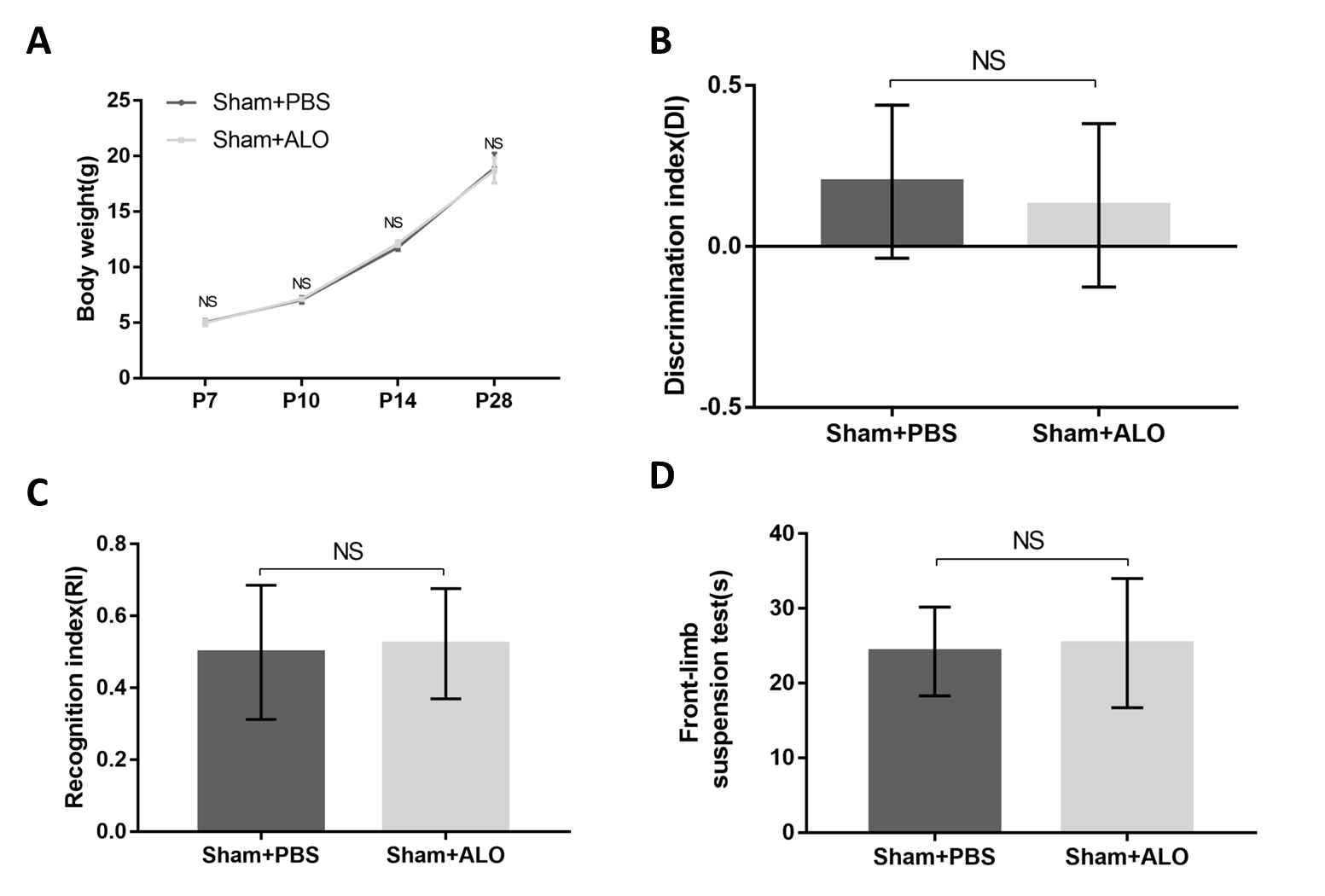

Supplement: Supplementary file 1 — Fig. S1. Effects of aloesin on normal mice. A. Changes in mouse body weight at different time points (n = 8 in each group). B. The discrimination index in the novel object recognition test (n = 8 in each group). C. The recognition index in the novel object recognition test (n = 8 in each group). D. Latency of mice to fall from the glass rod in the forelimb suspension test (n = 8 in each group). Statistical significance was determined by Student's t test. The values are expressed as the mean ± standard deviation: ns, p > 0.05. [file IID3-12-e1320-s001.tif]
